# Emerging Trends and Hot Spots in Hepatic Glycolipid Metabolism Research From 2002 to 2021: A Bibliometric Analysis

**DOI:** 10.3389/fnut.2022.933211

**Published:** 2022-07-12

**Authors:** Yanyu Zhou, Xiaoqi Lin, Suqing Yin, Ling Zhu, Yuting Yang, Yixuan Li, Baoshan Wang, Yingfu Jiao, Weifeng Yu, Po Gao, Liqun Yang

**Affiliations:** Department of Anesthesiology, Renji Hospital, Shanghai Jiao Tong University School of Medicine, Shanghai, China

**Keywords:** glycolipid metabolism, liver, bibliometric analysis, hot spots, CiteSpace

## Abstract

Glycolipid metabolic diseases, including type 2 diabetes, non-alcoholic fatty liver disease, obesity, hypertension, dyslipidemia, and atherosclerosis, which have become a major public health concern worldwide, are mainly triggered by *hepatic glycolipid metabolism* disorder. Bibliometric analysis has provided a comprehensive review of developments in *hepatic glycolipid metabolism* research and changes in research hotspots over the past 20 years. The articles regarding *hepatic glycolipid metabolism* from 2002 to 2021 were identified from the Science Citation Index-Expanded of Web of Science Core Collection. Acquired data were then processed by the CiteSpace software and the Online Analysis Platform of Literature Metrology to analyze trends and predict hot spots in this field. A total of 4,856 articles regarding *hepatic glycolipid metabolism* published from 2002 to 2021 were selected. The leading country was China. The Chinese Academy of Sciences was the most productive institution. Co-citation cluster labels revealed characteristics of ten main clusters: non-alcoholic fatty liver disease, gut microbiota, adiponectin, fructose, fgf21, fatty acid, liver x receptor, nr4a, obese mice, and bile acids. Keyword bursts analysis indicated that management, non-alcoholic fatty liver disease, and modulation were the newly emerging research hot spots. We described the overall structure of scientific research on *hepatic glycolipid metabolism* and presented systematic information to other researchers. The current focus on NAFLD and gut microbiota is critical to further study and will help explore effective therapeutic strategy for aberrant glycolipid metabolism in liver.

## Introduction

Liver plays a fundamental role in metabolic homeostasis and is the major site for synthesis, storage, and catabolism of glucose and lipid throughout the body (1). Hepatocyte is the place where glucose is catabolized into pyruvate *via* the glycolysis process, and then the pyruvate is utilized into fatty acids *via de novo* lipogenesis (2). It is reported that multiple factors participate in regulating hepatic glycolipid metabolic function, where the family of nuclear receptors (NRs) are also involved. The subtypes of NRs, such as LXRs, FXRs, and PPARs, are involved in homeostasis of glycolipid metabolism (3). Besides, independently of LXR, the SIRT6-AMPKα-mTORC1 signaling pathway regulates SREBP1c in liver, acting as a potential way to maintain *hepatic glycolipid metabolism* (4). It is reported that intestinal hypoxia-inducible factor 2α signaling is positively correlated with hepatic metabolic disorder (5), indicating the tight link between gut-liver axis and glycolipid metabolism homeostasis. Recent evidence has indicated that environmental pollutant, such as decabromodiphenyl ether, disrupts glycolipid metabolism through regulating the PI3K/AKT/GLUT4 pathway and the mTOR/PPARγ/RXRα pathway (6). Since people are accustomed to a sedentary and food-abundant lifestyle, glycolipid metabolism disorders have been highly globally prevalent, including non-alcoholic fatty liver disease (NAFLD) (7), insulin resistance (8), and diabetes (9). There have been a large number of studies on the development of novel drugs and treatment methods to improve glycolipid metabolism and then to relieve liver steatosis, liver fibrosis, insulin resistance, etc. Deletion of enzyme dihydroceramide desaturase 1, which disturbs ceramide double bond, could improve insulin resistance and hepatic steatosis (10). In addition, miRNA-mediated gene regulation promises to be a novel treatment of metabolic disease, for example, the miR-552-3p modulates the transcriptional activities of FXR and LXR to ameliorate *hepatic glycolipid metabolism* disorder (3). Moreover, the potential effect of traditional Chinese medicine on ameliorating type-2-diabetes is prevalent nowadays (11).

The global prevalence of glycolipid metabolism disorders has brought increased demand of efforts on in-depth research in this filed. Although glucose and lipid metabolic pathways are inseparable, there are few review articles summarizing the latest progress in this field and predicting research hot spots.

Bibliometric analysis is the timely and comprehensive review of publications during a specific period by analyzing their parameters, such as the number of publications, authors, countries and regions, references, keywords, and so on, which can provide an exhaustive overview of the intellectual landscape and make researchers cast light on the latest research trends (12). To our knowledge, no bibliometric study has been reported so far at the global level on *hepatic glycolipid metabolism*.

The aim of our study was to identify the publication trends and potentially significant hot spots on *hepatic glycolipid metabolism* by analyzing the records published from 2002 to 2021.

## Materials and Methods

### Data Sources and Search Strategies

**Figure 1:**
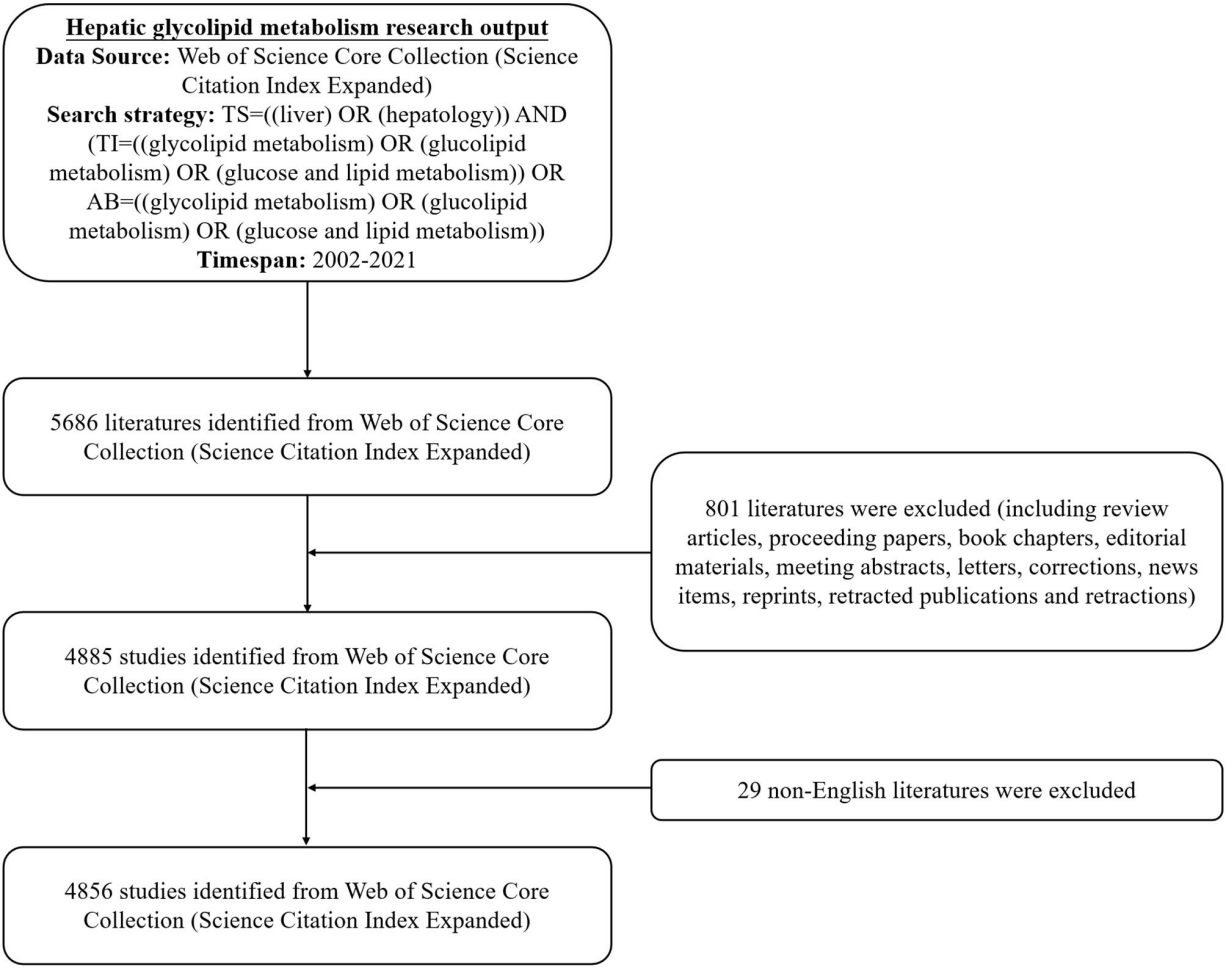
A flowchart for including and excluding publications.

The data of the study were extracted from the Web of Science Core Collection (WoSCC) for the period 2002 to 2021 on March 26, 2022. To avoid database update bias, we completed all data extraction and data downloads within the same day. We retrieved relevant publications through the following search strategy: TS = [(liver) OR (hepatology)] AND [TI = (glycolipid metabolism) OR (glucolipid metabolism) OR (glucose and lipid metabolism)] OR AB = [(glycolipid metabolism) OR (glucolipid metabolism) OR (glucose and lipid metabolism)] AND Language = English and Document type = Article. Only the Science Citation Index-Expanded (SCI-E) was selected. Then, the raw data were downloaded from WoSCC as text files involving full records. After the primary data search, two researchers (Yanyu Zhou and Xiaoqi Lin) screened all manuscripts individually to ensure they were all relevant to the subject of this study. In total, 4,856 articles were ultimately analyzed in our study. The detailed screening is shown in Figure 1.

### Bibliometric Online Platform Analysis

We processed the data systematically by Web of Science (https://wcs.webofknowledge.com) and the website of bibliometrics, the Online Analysis Platform of Literature Metrology (https://bibliometric.com/app). Web of Science was used to retrieve target data and to analyze the publication trend by year, and the data were imported into Excel 2016 to form a histogram. The website of bibliometrics, the Online Analysis Platform of Literature Metrology, was used to show the annual publication trend from different countries and regions, the inter-country/region cooperative relationships, and the top 10 most cited journals.

### CiteSpace Software Analysis

Full records and cited references of the retrieved articles were downloaded from the WoSCC database and saved as.txt format for further analysis by CiteSpace software (version 5.8 R3c). CiteSpace can output various kinds of indexes of significant difference, including temporal indexes, such as keyword burstness and structural indexes, such as betweenness centrality and silhouette value. Based on Freemans's betweenness centrality metrics (13), the centrality in CiteSpace means the degree of correlation of a node (e.g., one reference, one author) with other nodes. A node with high centrality is often seen as a key hub. The burstness of the frequency of an entity over time indicates a specific duration when an abrupt change in the frequency takes place, thus identifying emergent terms (14). Silhouette value is used to evaluate the quality of clusters created by CiteSpace using clustering algorithms. The silhouette value greater than.5 indicates that the objects lie well within their clusters. We set the following format: Time slicing from January 2002 to December 2021, years per slice choosing 1. The selection used a modified g-index in each slice: *g2* ≤ *k*Σ_*i*_ ≤ _*g*_*c*_*i*_, *k* ϵ *Z*^+^, *k* = *25*. CiteSpace was used to visualize the map of cooperation between countries/regions and between institutes, co-authorship, reference co-citation, and to figure out the bursts of keyword between 2002 and 2021. For keywords burst detection, the ones with little real significance, such as tissue and rat liver, were removed.

## Results

### Quantity and Trends Analysis of Published Papers

**Figure 2:**
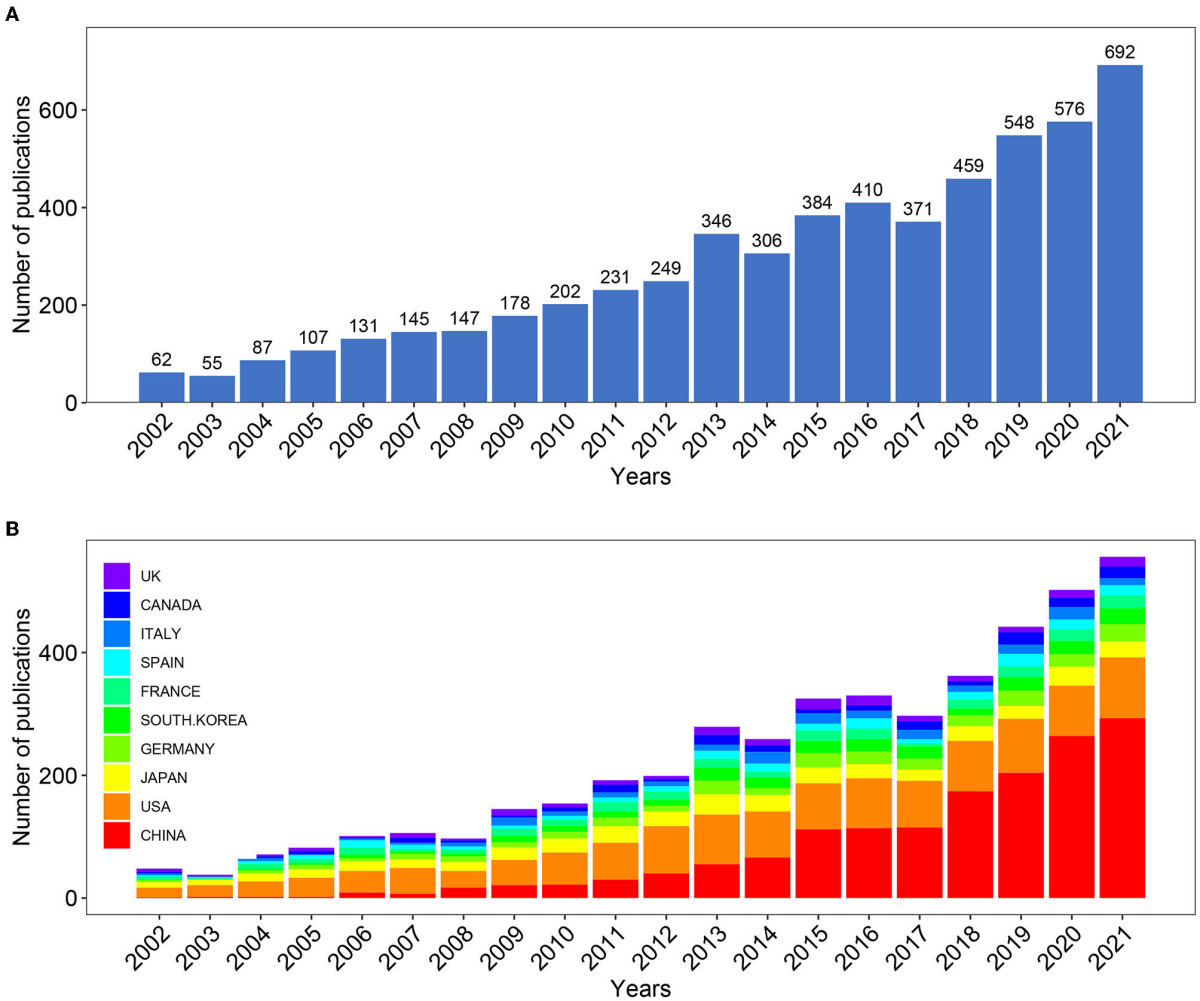
**(A)** Number of annual research publications and growth trends on the topic of hepatic glycolipid metabolism from 2002 to 2021, export of results from Web of Sciences. **(B)** Number of annual research publications and growth trends on the topic of hepatic glycolipid metabolism from 2002 to 2022, export of results from the Online Analysis Platform of Literature Metrology.

A total of 5,686 publications between 2002 and 2021 met the inclusion in the Web of Science database (SCI-E). We excluded 830 papers (non-English papers, review articles, proceeding papers, book chapters). As is shown in Figure 2A, studies on *hepatic glycolipid metabolism* can be roughly divided into two time periods. The publication trend of the early stage (2002-2016) maintained a fluctuated growth, while the number of publications in last the 5 years (2017-2021) grew at nearly triple the rate of the former, indicating that the *hepatic glycolipid metabolism* was getting more and more attention. Moreover, we used Microsoft Excel 2016 to build a growth trend model as follows: f(x) = ax^3^ + bx^2^ + cx + d, which predicted that nearly 1,000 articles will be published by 2025 (Supplementary Figure 1).

In order to figure out which countries or regions played leading roles in *hepatic glycolipid metabolism* filed during the past 20 years, the number of articles published by different countries and regions was counted in the website, the Online Analysis Platform of Bibliometrics (http://bibliometric.com/). The histogram showed the number of publications from the top 10 countries/regions over the 20 years (Figure 2B). Notably, the USA had long dominated the development of research on *hepatic glycolipid metabolism*, while the number of publications from China first exceeded that of the USA in 2015 and has maintained rapid growth in the last 5 years.

### Analysis of Intercountry/Region and Inter-institutional Cooperation

**Figure 3:**
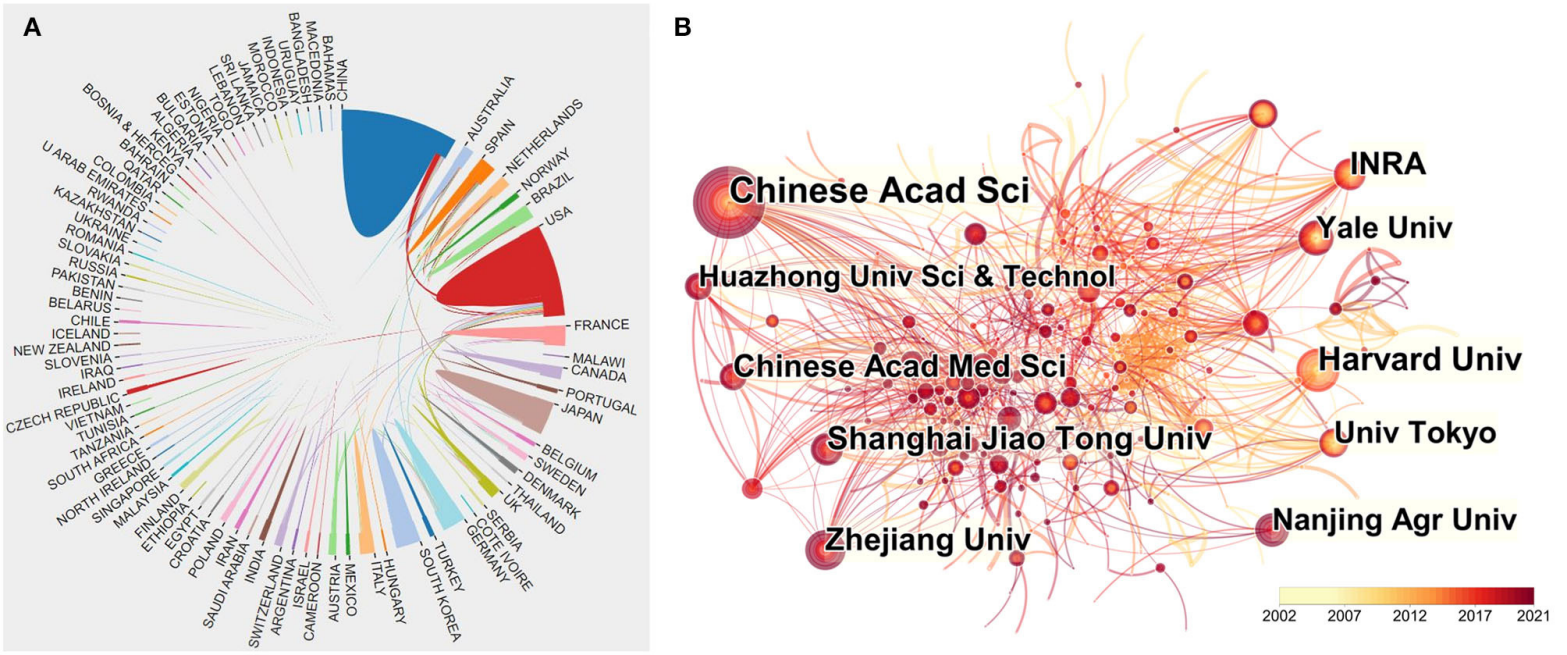
**(A)** Cooperative relationships between 82 countries/regions on the topic of hepatic glycolipid metabolism from 2002 to 2021. Data were exported from the Online Analysis Platform of Literature Metrology. **(B)** The CiteSpace network map of institutions involved in hepatic glycolipid metabolism research. The top 10 most productive institutions are shown. The size of the concentric circle represents the number of articles published by each institution, and the thickness of the connecting lines indicates the degree of cooperation between institutions. The darker the color of the concentric circle is, the more productive the institution has been on hepatic glycolipid metabolism in recent years.

Overall, the 4,856 articles were published by 82 countries and regions during 2002 to 2021. We analyzed cooperative relationships between these countries using the bibliometrics online analysis platform (Figure 3A). The result shows that the USA was the country that was the most frequently involved in international cooperation. Moreover, China and the USA had the most frequent cooperation among countries/regions.

A total number of 4,107 institutes made contributions to the hepatic glycolipid metabolism research. In order to clarify the inter-institutional cooperation in this field, we imported TXT format files into the CiteSpace software. As is shown in Figure 3B, the top 10 productive institutions were listed in the visualized graph, in which each concentric circle represented an institution, and the links indicated the strength of institutional cooperation with each other. The size of the concentric circle represented the number of articles published by each institution, and the thickness of the connecting lines indicated the degree of cooperation between institutions. The darker the color of the concentric circle is, the more productive the institution has been on *hepatic glycolipid metabolism* in recent years. Among them, Chinese Academy of Sciences published the most articles (99 articles) and has the largest centrality (0.17). Six of the top 10 most prolific institutions are from China, which implied the pivotal role of Chinese institutions in this field. Interestingly, a network density of only 0.0071 reflected the insufficient cooperation between institutions.

### Analysis of Co-authorship Network and Core Author Distribution

**Figure 4:**
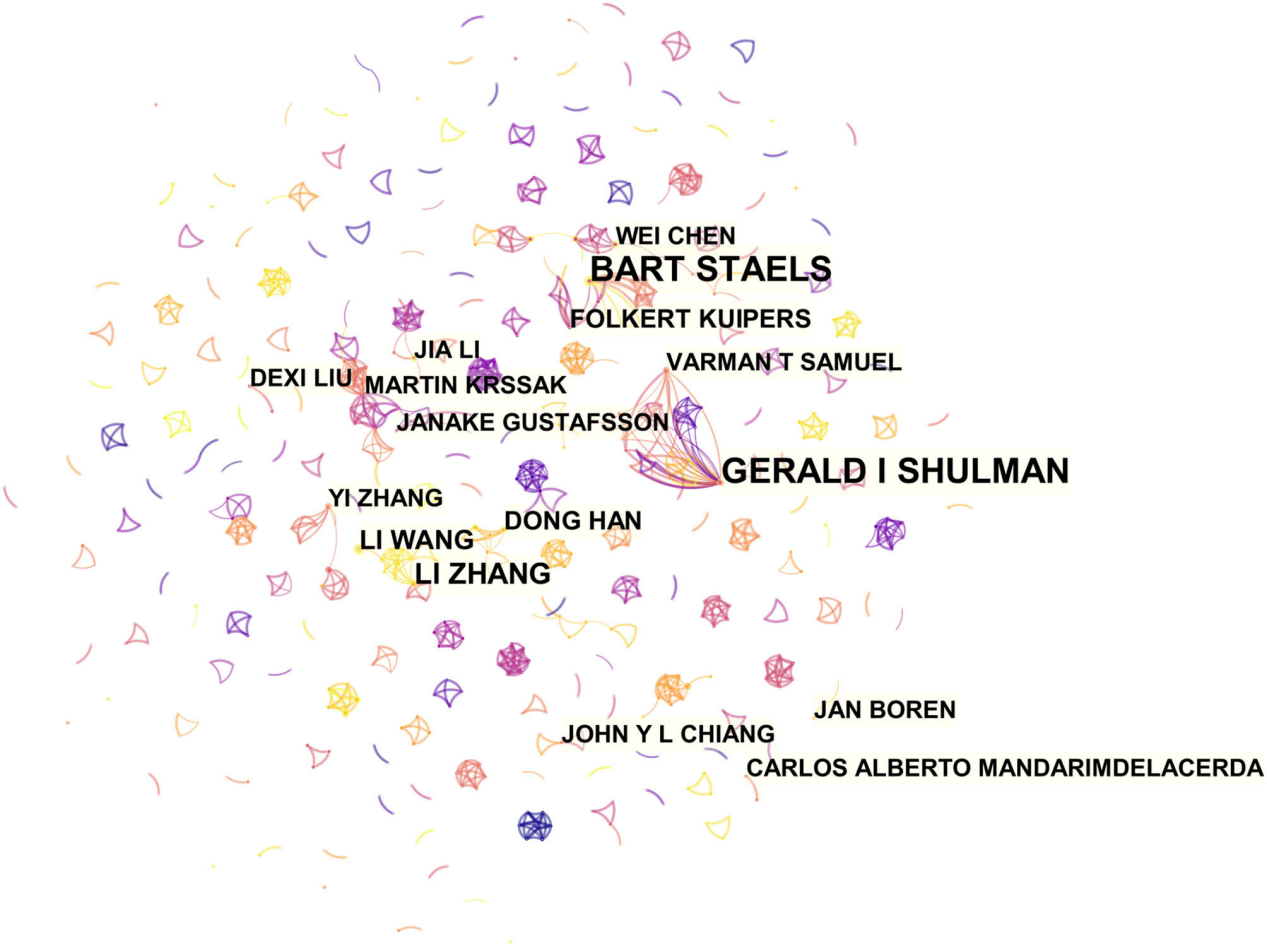
CiteSpace network of authorship in the field of hepatic glycolipid metabolism research. The top 16 authors with the most publications are shown. Each circle represents an author, and a link between two circles means a collaboration between each other.

About 24,756 authors made contributions to the publication outputs during the last 20 years, and the top 16 most productive authors are labeled in Figure 4. Since there were 10 authors who published 7 articles, 16 authors were displayed. The visual mapping provides vivid information to cooperative relationships, and, therefore, those help to figure out potential collaborators. Font size is positively associated with the number of articles published by certain authors. These authors are the most active when it comes to the *hepatic glycolipid metabolism* scientific community. Both Bart Staels from University of Lille and Gerald I Shulman from Yale School of Medicine published over 20 articles in this field during the two decades. The other most productive researchers related with *hepatic glycolipid metabolism* are also shown in Figure 4. Notably, different from the relatively independent network in institutions, most authors, especially the productive ones, preferred to build steady collaborative networks.

### Analysis of Journals

Over the past 20 years, 992 scholar journals have published a total number of 4,856 original articles. The bibliometrics online analysis platform was used to analyze journal influence. The top 10 most-cited journals related with *hepatic glycolipid metabolism* are presented in Table 1, which indicates that articles published in *Diabetes* were cited most frequently with 443 times during the past 20 years, followed by those in *Journal of Biological Chemistry* (320), *Journal of Clinical Investigation* (221), *Nature Medicine* (195), *PLoS One* (189), *Proceedings of the National Academy of Sciences of the United States of America* (186), *Endocrinology* (158), *Hepatology* (142), *Cell Metabolism* (141), and *American Journal of Physiology-Endocrinology and Metabolism* (136). Most strikingly, all of these journals are from the USA.

**Table 1:** The top 10 most active journals that published articles in hepatic glucolipid metabolism research (sorted by total citation).

**Rank**	**Journal title**	**Frequency**	**Total citations**	**Average citation per paper**	**Impact factor (2020)**	**Country**	**JCR**
1	Diabetes	86	443	5.15	9.461	USA	Q1
2	Journal of Biological Chemistry	68	320	4.71	5.157	USA	Q2
3	Journal of Clinical Investigation	22	221	10.05	14.808	USA	Q1
4	Nature Medicine	13	195	15.00	53.440	USA	Q1
5	PLoS One	171	189	1.11	3.240	USA	Q2
6	Proceedings of the National Academy of Sciences of the United States of America	30	186	6.20	11.205	USA	Q1
7	Endocrinology	64	158	2.47	4.736	USA	Q2
8	Hepatology	45	142	3.16	17.425	USA	Q1
9	Cell Metabolism	22	141	6.41	27.287	USA	Q1
10	American Journal of Physiology-Endocrinology and Metabolism	85	136	1.60	4.310	USA	Q1

### Analysis of Document Co-citation and Clustered Network

**Figure 5:**
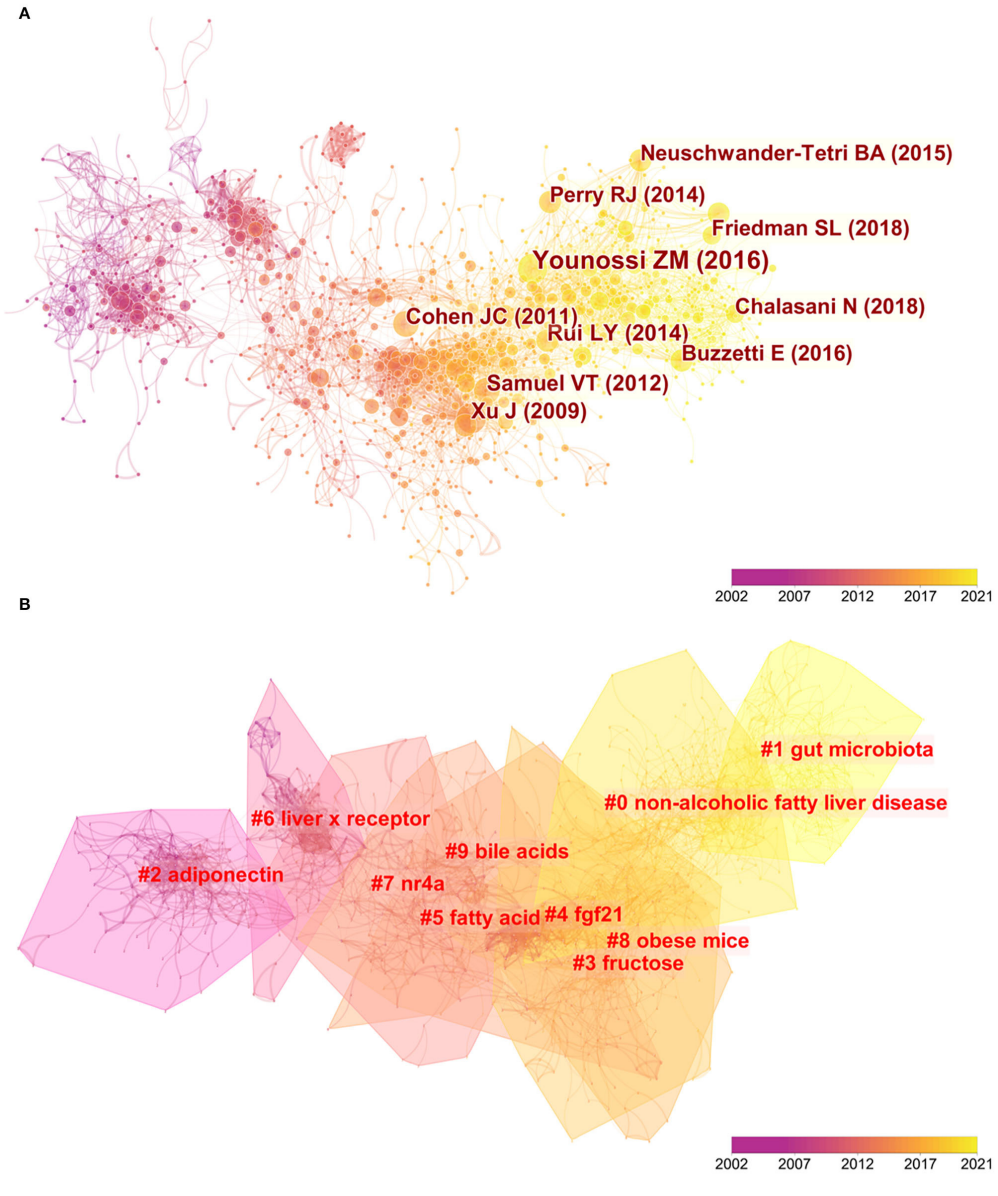
**(A)** A CiteSpace co-citation map of 157,691 references on hepatic glycolipid metabolism, a filter option showing the largest connected component only. Each node represents a reference, and the link between nodes means these articles were cited as references in the same article within the retrieved 4,856 articles. The size of node is positively related with the frequency of citation, and line thickness means the correlation with the co-cited papers. The yellower nodes represent that these papers have been frequently cited in recent years, while the redder ones represent those references cited in earlier years. **(B)** Clustered networks of co-citation status of the investigated reference and the 4,856 citing articles via CiteSpace. The top 10 largest clusters of citing articles are shown.

Document co-citation is a method to clarify literature co-cited by a group of authors. Namely, this method is used to evaluate the relationship of two documents by visualizing their co-occurrence of citations (15). From 2002 to 2021, a total of 4,856 articles and their 157,691 references (excluding self-citations) retrieved from WoSCC were analyzed by CiteSpace to find out mutual homogeneity and then to cluster them. A map of co-citation reference in CiteSpace on *hepatic glycolipid metabolism* research is presented in Figure 5A. Each node represents a reference, and the link between nodes means these articles were cited as references in the same article within the retrieved 4,856 ones. The size of node is positively related with the frequency of citation, and line thickness means the correlation with the co-cited papers. Moreover, the yellower nodes represent that these papers have been frequently cited in recent years, while the redder ones represent those references cited in earlier years. The top ten articles sorted by frequency of citations are presented in Table 2.

**Table 2:** The top 10 high-cited papers in the hepatic glucolipid metabolism field during 2002 to 2021.

**Rank**	**Title**	**First author**	**Journal**	**Year**	**Cited frequency**	**DOI**
1	Global epidemiology of nonalcoholic fatty liver disease-Meta-analytic assessment of prevalence, incidence, and outcomes	Younossi ZM	HEPATOLOGY	2016	62	10.1002/hep.28431
2	Energy metabolism in the liver	Rui LY	COMPR PHYSIOL	2014	37	10.1002/cphy.c130024
3	Mechanisms for insulin resistance: common threads and missing links	Samuel VT	CELL	2012	34	10.1016/j.cell.2012.02.017
4	Human fatty liver disease: old questions and new insights	Cohen JC	SCIENCE	2011	34	10.1126/science.1204265
5	Fibroblast growth factor 21 reverses hepatic steatosis, increases energy expenditure, and improves insulin sensitivity in diet-induced obese mice	Xu J	DIABETES	2009	34	10.2337/db08-0392
6	The diagnosis and management of nonalcoholic fatty liver disease: Practice guidance from the American Association for the Study of Liver Diseases	Chalasani N	HEPATOLOGY	2018	32	10.1002/hep.29367
7	Mechanisms of NAFLD development and therapeutic strategies	Friedman SL	NAT MED	2018	32	10.1038/s41591-018-0104-9
8	The role of hepatic lipids in hepatic insulin resistance and type 2 diabetes	Perry RJ	NATURE	2014	31	10.1038/nature13478
9	Farnesoid X nuclear receptor ligand obeticholic acid for non-cirrhotic, non-alcoholic steatohepatitis (FLINT): a multicentre, randomized, placebo-controlled trial	Neuschwander-Tetri BA	LANCET	2015	31	10.1016/S0140-6736(14)61933-4
10	The multiple-hit pathogenesis of non-alcoholic fatty liver disease (NAFLD)	Buzzetti E	METABOLISM	2016	31	10.1016/j.metabol.2015.12.012

Results showed that the highest-ranking cited reference was a meta-analysis review published by *Hepatology* in 2016 (16). Researchers retrieved and analyzed a total number of 8,515,431 patients from the published studies from 1989 to 2015 in order to determine the prevalence, incidence, risk factors, and long-term outcomes of patients with non-alcohol lipid liver disease (NAFLD). The second-ranked paper was also a review published by Comprehensive Physiology in 2014 (17). On the contrary, this review focused on the metabolic process of glucose and fatty acid and related diseases (obesity, NAFLD, and type 2 diabetes) caused by their dysfunction. The third-ranked paper discussed the molecular mechanism of insulin resistance, a complex metabolic disorder caused by overload of accumulation of lipid metabolites in liver and skeletal muscle (18). All these three reviews discussed pathological and physiological processes of *hepatic glycolipid metabolism* clinically and molecularly. Since literature cited by papers is considered as the opinion that the author intends to express, the most cited papers listed in Table 2 made great contributions to *hepatic glycolipid metabolism* research and could be seen as most recognized in this field.

The map of co-citation clustered according to keywords generated from the references of 4,856 citing articles by CiteSpace is shown in Figure 5B. The analysis of co-citation clusters revealed the most relevant terms on *hepatic glycolipid metabolism* research by the way of hierarchical cluster labels, which included #0 non-alcoholic fatty liver disease, #1 gut microbiota, #2 adiponectin, #3 fructose, #4 fgf21, #5 fatty acid, #6 liver x receptor, #7 nr4a, #8 obese mice, and #9 bile acids. The number of cluster tags is reversely correlated with the number of articles that each cluster included. In other words, the cluster marked as the #0 contains the largest number of papers among the 157,691 co-cited references. Moreover, the yellower the color patch to which each cluster belongs, the more frequently the references in this cluster have been cited in recent years (Figure 5B). A summary of clusters is listed in Table 3.

**Table 3:** A summary of 10 clusters.

**Cluster ID**	**Top term**	**Size**	Silhouette^*^
0	Non-alcoholic fatty liver disease	197	0.853
1	Gut microbiota	130	0.932
2	Adiponectin	121	0.892
3	Fructose	118	0.87
4	fgf21	107	0.93
5	Fatty acid	97	0.896
6	Liver x receptor	74	0.972
7	nr4a	65	0.858
8	Obese mice	65	0.915
9	Bile acids	58	0.953

* *Silhouette value >0.5 means the clustering results are reliable*.

### Analysis of the Research Trend and Burst Detection With Keywords

**Figure 6:**
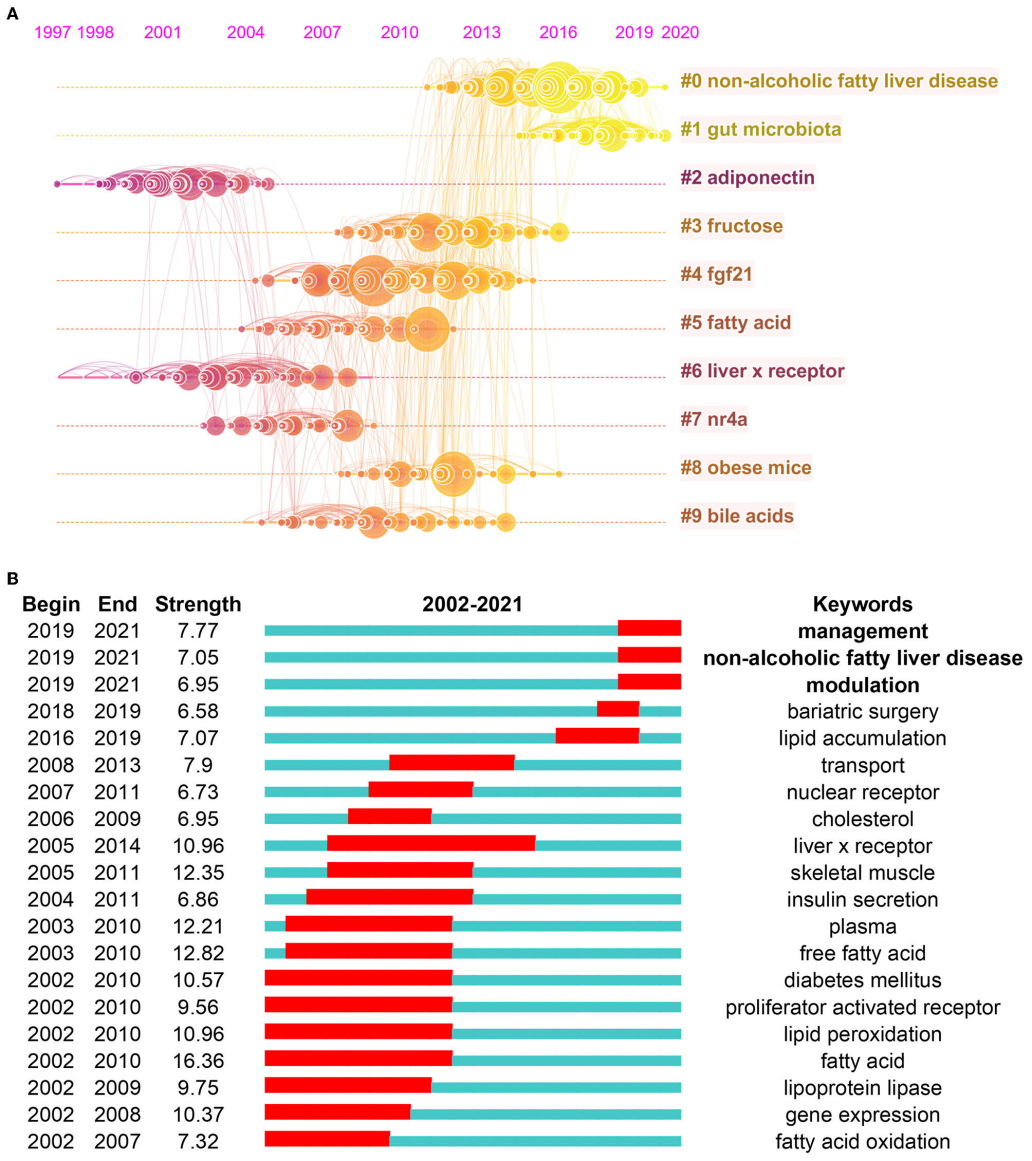
**(A)** A timeline view of the top 10 largest clusters of citing articles in the field of hepatic glycolipid metabolism. Right side = cluster labels. **(B)** Keywords with the strongest burst strength of the 4,856 citing articles on hepatic glycolipid metabolism research between 2002 and 2021. Keywords marked in red indicate a sudden increase in usage frequency of this keyword during that period. Blue represents a relatively unpopular time period.

In order to clearly describe the evolution of hot spots in *hepatic glycolipid metabolism* in the past 20 years, a timeline view is displayed in Figure 6A. As is shown, each circle represents a main cited paper in the certain cluster, and the citation tree-rings of different sizes on timeline represent citation rates. We found that, in the *hepatic glycolipid metabolism* field, adiponectin and liver x receptor were hot spots, which started in 1997 and ended before 2010. The clusters of non-alcoholic fatty liver disease starting in 2011 occupied the highest degree of citation bursts till 2020, followed by gut microbiota. The focus of research in *hepatic glycolipid metabolism* seems to have been transferred from adiponectin and liver x receptor to non-alcoholic fatty liver disease and gut microbiota.

Keyword burst detection was another method that helps to catch research hot spots quickly. Figure 6B displays the top 20 references with the strongest keyword bursts on *hepatic glycolipid metabolism* research during the period of 2002-2021. The blue line indicates the time range of 2002-2021, and the red line indicates the period that the burst maintains. Among them, the keyword bursts by the end of 2021 were led by management with a strength of 7.77, non-alcoholic fatty liver disease with a strength of 7.05, and modulation with a strength of 6.95. The keyword management and modulation described the integrated and diverse application of novel drugs and treatments to glycolipid metabolic disorders. The keyword non-alcoholic fatty liver disease indicated the major disease of glycolipid metabolic disorders.

## Discussion

For this bibliometric analysis study, we found 4,856 articles regarding *hepatic glycolipid metabolism* research from 2002 to 2021 in the Web of Science database (SCI-E). The overall publication number maintained a stable growth trend, especially from the year 2017, the number of papers in this field presented a booming growth, so there were a variety of research contents. Through the online bibliometric analysis platform and CiteSpace software, our study analyzed publication trends about *hepatic glycolipid metabolism* from all aspects, has demonstrated a systematic view of this field over the past two decades, and provided guidance for future studies. Therefore, with the help of our bibliometric analysis, researchers who are interested in *hepatic glycolipid metabolism* can easily have a general understanding and quickly grab the latest research hot spots.

The outputs of annual publications in this field have indicated a continuous and steady upward trend in the last two decades. Notably, the increasing rate got even faster from 2017, indicating that the study of metabolism in liver has remained widely prevalent. The Figure 2B shows that the USA and China were the two leading countries that significantly contributed to *hepatic glycolipid metabolism* research. On the one hand, the number of publications from the USA has kept a certain proportion over the two decades. On the other hand, although Chinese researchers emerged in this field later than other countries, undoubtedly, they have acted as a leading role and made great contributions to *hepatic glycolipid metabolism* research. Moreover, enhancing international cooperation has become an irreversible trend, and this kind of cooperation mode is more conducive to the output of high-quality research results. As for the collaborations between countries/regions shown in Figure 3A, China still ranked first in terms of the cooperative relationship with other countries/regions, especially with the USA. Besides, six of the top 10 institutes ranked by the publication count are from China, and their contributions to this field have been mostly concentrated in recent years (Figure 3B). Therefore, these results demonstrated Chinese researchers' deep exploration and great potential scientific innovation in the *hepatic glycolipid metabolism* field.

Among the top 10 most cited journals, the journal *Diabetes* possessed the most citations (443), and it is a journal associated with physiology and pathology of diabetes, which is an important component in glycolipid disorders. Besides, the papers published in *Nature Medicine* occupied the most average citation per paper, and its population was probably attributed to the high impact factor and the great influence that came with it. The journal *PLoS One* had the largest publication count (171 papers), while its average citation per paper was 1.11 times, which implied the papers published on *PLoS One* may be of relatively low quality and of little citation value. All of the ten journals were from the USA, reflecting that this country provided an important platform for the development of the *hepatic glycolipid metabolism* field.

CiteSpace is used to summarize and analyze networks of co-cited references and keywords based on bibliographic records extracted from the Web of Science (19), which can help figure out emerging trends in the future and find potential hot spots on *hepatic glycolipid metabolism*. Keyword burst means the keywords are significantly cited by papers over a period of time, and it is considered as another important indicator of study hot spots or emerging trends over time (20). As is shown in Figure 6B, top 20 keyword bursts with the strongest citation are listed, revealing potential hot spots on *hepatic glycolipid metabolism* over the last two decades. Notably, most bursts of these keywords listed in the table began in 2002 and did not continue till the end of 2021, indicating that such research trends reflected by some keywords were prevalent, while they cannot be considered as current hot spots of this field. Among them, the keyword burst, management, began in 2019 and lasted until the end of 2021 and ranked first, with a strength of 7.77. Articles related with this keyword focused on new drug management and treatment of glycolipid metabolic disorders. Liver function monitoring and liver disease prevention are of vital importance on patients (21). Researchers tended to explore comprehensive treatments from multiple perspectives, including human umbilical cord-derived mesenchymal stem cells (22), obeticholic acid (23), and selenium-enriched Bifidobacterium longum DD98 (24).

Next, the citation burst of keyword non-alcoholic fatty liver disease, which has lasted until the end of 2021 is worthy of great importance. In our study, co-citation visualization analysis indicated that over half of the top 10 most frequently cited papers carried out research around all aspects of NAFLD, including pathology, diagnosis, epidemiology, and molecular mechanisms et al. Interestingly, these papers were published after 2011, and this was consistent with the timeline of research interest in NAFLD in Figure 6A. NAFLD is the most common chronic liver disease around the world, which is closely related with obesity, type 2 diabetes mellitus, and other metabolic disorder (25). Not only western countries, but even Asian countries have developed a sedentary lifestyle, and more and more people experience overnutrition. The prevalence of obesity increases burden of NAFLD significantly (26). Consistent with the keyword burst, there were six literatures' topics closely associated with NAFLD in the top 10 most cited references. The highest-ranking cited reference was a meta-analysis review, and it analyzed the prevalence, incidence, risk factors, and long-term outcomes of patients with NAFLD (16). The second most cited reference was a review article focusing on the metabolic process of glycolipid metabolism and related disorders, such as NAFLD (17). The citation burst of references related with the topic of NAFLD reflected the vital importance of in-depth study on this glycolipid disorder. With the continuous growth of incidence, every aspect of NAFLD is under thorough research. Therefore, more breakthroughs will surely be made in future studies (27).

The third keyword burst, which lasted until the end of 2021, was modulation. The related articles focused on molecular mechanism of *hepatic glycolipid metabolism* from all aspects. It is reported that caveolin-1, a structural protein of caveolae involved in lipid homeostasis, played an important role in modulation of lipid utilization for liver regeneration (28).

By the way, we found that the cluster, gut microbiota, sprung up from about 2014 and kept popular till the end of 2021. Gut microbiota is a central regulating factory of human metabolism, whose composition and function are different because of people's daily diet structure (29). Both type 2 diabetes and obesity are reported closely related with gut microbiota dysbiosis, and these are important inducements of NAFLD (30).

Overall, these results are inseparable from the occurrence and development of diseases caused by aberrant glycolipid metabolism in the liver and reflect an impact on future hot spots, leading the way for the following research.

Our study has some limitations. Firstly, the data analyzed were only extracted from the SCI-E database from WoSCC, and records from other important search engines, such as PubMed, Embase, and Ovid, were excluded, resulting in the insufficient representation of the publications on *hepatic glycolipid metabolism* over the past 20 years by the collected data. However, the data retrieved from WoSCC included comprehensive records like title, author, institute, and reference, which are necessary for bibliometric analysis. Besides, only the data retrieved from WoSCC rather than other databases include complete information, such as references, so that co-citation analysis can be completed by CiteSpace. Secondly, in that English is still the preferred language for academic journals today, our study merely focused on papers published in English, causing omission of the articles published in other languages.

## Conclusion

In conclusion, with in-depth research in various fields and the growing number of relevant papers, the need for a quick grasp overview and the latest progress grows then. We took the example of the *hepatic glycolipid metabolism* field and described the overall structure of scientific research with vivid and systematic information to other researchers by CiteSpace and the Online Analysis Platform of Literature Metrology. Moreover, the current focus on gut microbiota, NAFLD, and comprehensive management of treatment for metabolic disorders is critical to further studies and helps to explore effective therapeutic strategy for aberrant glycolipid metabolism in liver.

## Data Availability Statement

The original contributions presented in the study are included in the article/Supplementary Material, further inquiries can be directed to the corresponding author/s.

## Author Contributions

LY and PG raised the conception of the study and designed the study. YZ and XL searched and screened articles, extracted data, and drafted the manuscript. SY, LZ, YY, YL, and BW helped interpret the data for the work. LY, PG, YJ, and WY revised the manuscript and edited critically. All the authors contributed to the article and approved the submitted version.

## Funding

This study was funded by Shanghai Hospital Development Center (No. SHDC2020CR2055B), Science and Technology Commission of Shanghai Municipality (No. 20410760500), the National Key Research and Development Program (No. 2018YFC201803), Medical Engineering Cross Research Foundation of Shanghai Jiao Tong University (No. YG2022QN022), Key Specialty Construction Project of Pudong Health and Family Planning Commission of Shanghai (No. PWZXQ2017-06), Shanghai Municipal Key Clinical Specialty (No. shslczdzk03601), Innovation Program of Shanghai Municipal Education Commission (No. 2019-01-07-00-01-E00074), and Shanghai Engineering Research Center of Perioperative Organ Support and Function Preservation (20DZ2254200).

## Conflict of Interest

The authors declare that the research was conducted in the absence of any commercial or financial relationships that could be construed as a potential conflict of interest.

## Publisher's Note

All claims expressed in this article are solely those of the authors and do not necessarily represent those of their affiliated organizations, or those of the publisher, the editors and the reviewers. Any product that may be evaluated in this article, or claim that may be made by its manufacturer, is not guaranteed or endorsed by the publisher.
