# Supplementary material for: Emerging Trends and Hot Spots in Hepatic Glycolipid Metabolism Research From 2002 to 2021: A Bibliometric Analysis
Source: Front Nutr. 2022 Jul 12;9:933211. doi: 10.3389/fnut.2022.933211 (PMC9326119; doi:10.3389/fnut.2022.933211)
Supplement: Supplementary file 1 [file Image_1.pdf]

frontiers

Supplementary Material

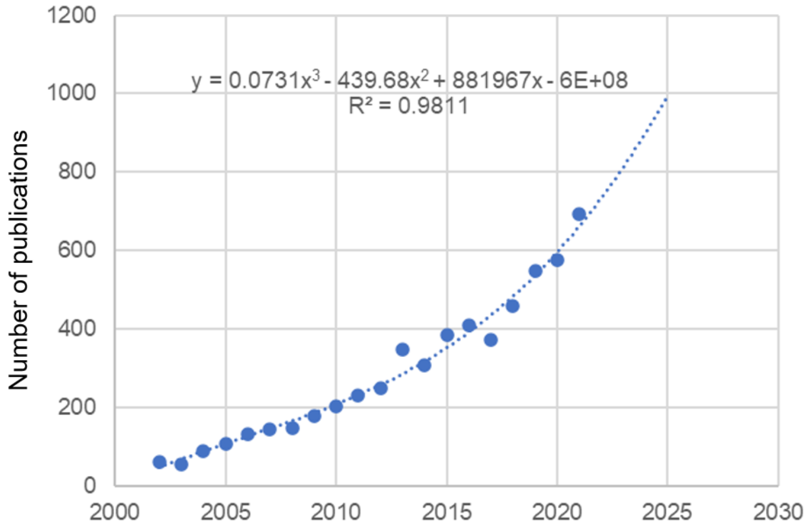

Supplementary Figure 1. Output of publications and growth prediction of *hepatic glycolipid metabolism* research. The number of publications from 2002 to 2021 are presented by large scatters. The dashed line represents the predicted curve, R^2=0.9811.
